# Untold Stories of Black and Racialized Immigrants with Disabilities During COVID-19 in the Greater Toronto and Hamilton Area

**DOI:** 10.3390/healthcare14020205

**Published:** 2026-01-14

**Authors:** Chavon Niles, Karen Yoshida, Kelsey Vickers, Jheanelle Anderson, Yahya El-Lahib, Rana Hamdy, Nadeen Al Awamry

**Affiliations:** 1 Department of Physical Therapy, Temerty Faculty of Medicine, University of Toronto, Toronto, ON M5S 1A8, Canadarana.hamdy@mail.utoronto.ca (R.H.); nadeen.alawamry@mail.utoronto.ca (N.A.A.); 2 Independent Researcher, Toronto, ON M5G 1V7, Canada; 3 Faculty of Social Work, University of Calgary, Calgary, AB T2N 1N4, Canada

**Keywords:** health equity, critical disability studies, critical race theory, accessibility, rehabilitation, community care, COVID-19

## Abstract

**Background**: Black and racialized immigrants with disabilities in Canada face overlapping systems of exclusion rooted in racism, ableism, and migration status. Yet, their experiences within health and rehabilitation services during the COVID-19 pandemic remain largely undocumented. This study explores how structural inequities shaped access to healthcare, rehabilitation, information, and community supports in the Greater Toronto and Hamilton Area (GTHA). **Methods**: Using narrative inquiry, ten in-depth interviews were conducted with participants who identified as Black or racialized, disabled, and having immigrated to Canada within the last 10 years. Narratives were analyzed through reflexive thematic analysis to identify how systems, relationships, and policies interacted to shape daily life, health and rehabilitation navigation during the pandemic. **Results**: Participants described systemic barriers in health and rehabilitation systems, experiences of “othering” and conditional belonging, and the critical role of informal and faith-based networks in navigating inaccessible services. Pandemic policies often intensified existing inequities. **Conclusions**: Findings underscore the need for intersectional health and rehabilitation planning that centers the voices of Black and racialized disabled immigrants. Addressing systemic racism and ableism is essential for equitable preparedness in future public health emergencies.

## 1. Introduction

The COVID-19 pandemic amplified long-standing inequities within health, rehabilitation, and social systems. These inequities underscore how racism, ableism, xenophobia, and colonial legacies structure people’s experiences of health, illness, and disability [1,2]. Disabled immigrants were made vulnerable through policies, practices, and attitudes that excluded them from timely information, income supports, and healthcare [3,4]. Globally, disabled people experienced heightened risks of infection and disruptions in essential health and rehabilitation services because of ableist assumptions embedded in emergency planning and triage systems [5,6]. The pandemic was a health and social crisis that further exposed the fragility of healthcare systems and the deep-rooted inequities that determine who is considered worthy of protection and whose needs can be deferred [7,8].

Although research on COVID-19 and disability has expanded, much of it continues to focus on biomedical outcomes, long COVID, and individual health behaviours rather than the social, political, and economic conditions that sustain inequity [7,9]. Less attention has been given to how racism, xenophobia, and ableism intersect to influence immigrants’ and refugees’ experiences of disability, health and rehabilitation [2,3,4]. These interlocking systems determine who is welcomed, resourced, or excluded within healthcare and social-support structures. In Canada, immigrants represent about one-fifth of disabled people, yet their experiences, particularly those of Black and racialized immigrants, remain largely absent from both research and public-health and rehabilitation planning [10,11,12].

Emerging scholarship in Canada has begun to highlight how pandemic responses compounded these inequities. Yoshida et al. [3] describe how COVID-19 amplified the complexity of disability and race, revealing how public-health measures often ignored the realities of Black and racialized disabled people and excluded them from decision-making processes. Building on this, Niles et al. [7] applied a disability justice lens to examine Ontario’s COVID-19 response, showing how government definitions of “essential” work and “vulnerable” populations reproduced hierarchies of value that marginalized disabled communities. Similar patterns of exclusion and policy neglect have been documented globally [4,6]. Both Canadian and international studies underscore that inequities during the pandemic were a predictable outcome of systems that tie access and protection to productivity and compliance.

The Greater Toronto and Hamilton Area (GTHA) was an early epicentre of COVID-19 in Ontario, Canada and a clear example of these inequities [13]. Government responses that relied on digital platforms, written English materials, and ableist assumptions about technology excluded many communities from critical public-health information. For Black and racialized immigrants with disabilities, these barriers were intensified by discriminatory immigration systems, fragmented and inaccessible health and rehabilitation pathways and economic precarity rooted in colonial histories [2,7,12]. These experiences echo the findings of Underwood et al. [1], who show how procedural ableism, expressed through policies and processes that appear neutral, systematically excludes disabled people from education, childcare, and social services. They also align with national analyses calling for equity-driven indicators of preparedness and accountability in future emergencies [14].

This study is grounded in the recognition that inequities are maintained through everyday systems of governance. It draws on critical approaches that trace how disability and race are socially and politically constructed through institutional norms and policy design [15,16,17]. These frameworks foreground the power relations that shape how health and rehabilitation services are organized, valued, and restricted.

The purpose of this study is to examine the experiences of Black and racialized immigrants with disabilities in the GTHA, focusing on how structural inequities shaped their interactions with health and rehabilitation systems during the COVID-19 pandemic.

This study also highlights the resistance and community care practices that emerge within oppressive systems. It builds on previous work documenting the compounded impacts of ableism, racism, and migration status in Ontario [3,7], extending those insights through stories that capture how these intersecting systems are lived in health and rehabilitation contexts. This paper contributes to a growing body of research calling for accessible, anti-racist, and culturally responsive public-health and rehabilitation planning to ensure that future emergency responses address, rather than reproduce, the inequities experienced during COVID-19 [8,14].

Recent national data reinforce these findings. A 2022 Statistics Canada Health Report comparing individuals with and without chronic conditions found that nearly one-third (32.0%) of those with at least one chronic condition had a medical appointment cancelled, rescheduled, or delayed because of COVID-19, compared with 24.2% of those without chronic conditions [18]. Individuals who identified as immigrants, women, or persons with disabilities were also more likely to have delayed contacting a healthcare provider because of fear of exposure to the virus [12,14]. These patterns point to the structural nature of access barriers in Canada and the ways that intersecting forms of oppression, including ableism and immigrant status, compound vulnerability during public-health emergencies. Such evidence underscores the importance of examining how health and rehabilitation systems respond to the needs of Black and racialized immigrants with disabilities beyond quantitative indicators of access.

This study is grounded in Critical Disability Studies and Critical Race Theory. Together, these frameworks view disability and race as social and political constructs produced through systems of power. They reveal how ableism, racism, and xenophobia intersect in health and rehabilitation systems, determining whose needs are recognized and whose are neglected. Using these frameworks, we examine participants’ stories as evidence of how policy, practice, and discourse reproduce inequities and resistance.

### 1.1. Background

Scholars and advocates have shown that disabled people were not merely “left behind” during the pandemic but were excluded through design decisions that prioritized institutional efficiency over accessibility [2,5,19]. Across jurisdictions, public-health measures relied on narrow biomedical logics that discounted the social, cultural, and relational dimensions of wellbeing. As Barden and Bê [20] note, the pandemic simultaneously created new possibilities for connection through digital platforms and renewed exclusions that deepened inequities. Similarly, van Holstein, Wiesel, Bigby, and Gleeson [6] describe how people with intellectual disabilities experienced “more-than-care” relationships during lockdowns, revealing how community networks became vital infrastructures of support when formal systems failed.

Global disability research has reframed the COVID-19 pandemic as both evidence of systemic neglect and an opportunity to reimagine more inclusive, people-centred systems. Within disaster studies, Kett, Cole, and Twigg [21] demonstrate that exclusion is often produced through governance structures that prioritize centralized decision-making while overlooking local knowledge, interdependence, and community networks. Their work provides a critical foundation for understanding how emergency planning can marginalize disabled people by failing to recognize informal systems of support. COVID-19-specific disability scholarship builds on these insights, showing how top-down pandemic responses reproduced inequities when formal health, rehabilitation, and social systems became inaccessible. Findings from the Global South reinforce this point. Juvva, Nayar, and Sinha [4] show that during India’s lockdown, people with disabilities relied on local networks to navigate inaccessible systems, while Ned, McKinney, McKinney, and Swartz [22] document similar dynamics in South Africa, where social relations became the foundation of survival. These patterns closely align with participants’ accounts in this study, who described developing informal networks of translation, transportation, and emotional support when formal systems faltered.

Oguamanam [8] argues that existing human-rights instruments, such as the United Nations Convention on the Rights of Persons with Disabilities, were not operationalized in pandemic responses. This policy gap allowed states to overlook accessibility obligations and to implement triage and containment measures that undermined disabled people’s autonomy and participation. His analysis aligns with Caleb and Gallin [2], who demonstrate how exclusionary public-health laws in the United States privileged institutional convenience over human rights, and with Stienstra [19], who situates these failures within broader global inequities in disability policy. Situating rehabilitation within this rights-based context underscores that access to equitable care is not a matter of service provision alone but a question of justice and governance.

Connon et al. [23] describe the pandemic as a “social disability disaster,” in which social relations, not pathogens, produce disablement. Their analysis shows how isolation, fear, and bureaucratic rigidity reshape the experience of disability itself. This perspective helps interpret the disruptions in rehabilitation and community care documented in this study, where policies intended to protect health often generated new forms of exclusion. As Juvva et al. [4] and van Holstein et al. [6] also show, exclusion often coexists with creativity, as communities generate new forms of interdependence and resistance. 

Taken together, these perspectives reveal that people-centred systems depend on interdependence, shared accountability, and cultural responsiveness. Barden and Bê [20] call for moving beyond “care” as an individualized transaction toward practices of solidarity that value lived experience as expertise. In health and rehabilitation systems, this shift requires recognizing that health and participation are co-produced through relationships among service users, providers, and communities. These insights underscore the need for narrative approaches that attend to lived experience as knowledge that guides the development of equitable, community-driven health and rehabilitation systems capable of withstanding future public-health emergencies [12,14].

### 1.2. Ethical Considerations

This study was conducted in accordance with the Declaration of Helsinki, and the protocol was approved by the University of Toronto Research Ethics Board #44666 on 18 July 2023. Participants received detailed study information and provided written consent before participation. To acknowledge their time and expertise, each participant received an honorarium. All identifying information was removed from transcripts, and audio files were stored securely on encrypted servers accessible only to the research team.

## 2. Materials and Methods

### 2.1. Study Design

#### Theoretical Framework

This study is grounded in Critical Disability Studies (CDS) and Critical Race Theory (CRT), which together provide a framework for examining how systems of power shape access to health and rehabilitation systems. Both perspectives reject the idea that disability or race are individual characteristic. Instead, they understand them as social and political constructs produced through historical, institutional, and policy contexts.

CDS shows us how disability is not a deficit located in the body but a reflection of how environments, norms, and policies define what is “normal” and “functional” [24,25]. CDS directs attention to how rehabilitation, education, and employment systems uphold normative standards of ability and measure inclusion by proximity to that norm. This framework encourages us to ask how systems must transform to reflect the realities of disabled lives.

CRT foregrounds how racism, xenophobia, and colonial histories shape whose bodies and voices are considered credible within these same systems [15,16]. It situates health and rehabilitation within broader structures that value whiteness, citizenship, and economic productivity. CRT reveals how practices that appear neutral, such as intake forms, coverage policies, and communication norms, are in fact designed around particular racial and cultural assumptions. 

Bringing these frameworks together allows for a deeper understanding of how ableism and racism operate simultaneously in health and rehabilitation systems. They expose how access to services and credibility are organized through intersecting systems of oppression.

This study used a narrative inquiry approach to explore how Black and racialized immigrants with disabilities in the GTHA experienced health and rehabilitation services during the COVID-19 pandemic. Narrative inquiry was chosen because it privileges people’s lived experiences and the meanings they attach to them [16,26,27,28]. It allows participants to recount how social, political, and institutional conditions shape their everyday realities.

### 2.2. Participant Recruitment

We used convenience sampling to recruit participants between August and November 2023. Recruitment occurred through community and health organizations serving immigrants, refugees, and people with disabilities in the GTHA. A digital recruitment flyer was circulated through these organizations and shared on social media platforms, including Instagram, Twitter, and LinkedIn. Interested individuals contacted the lead author (C.N.) by email and were invited to a Zoom-based information session. During this session, the study objectives were reviewed, questions were addressed, and eligibility criteria were confirmed. Individuals who met the inclusion criteria and expressed interest in participating were put on an inclusion list. Eligibility criteria included:

1. Self-identifying as Black or racialized;
2. Identifying as a person with a disability/disabilities (including physical, sensory, cognitive, or episodic);
3. Having immigrated to Canada within the last 10 years;
4. Residing in the GTHA during the COVID-19 pandemic;
5. Comfortable communicating in English.

Ten participants were enrolled, reflecting a range of ages, different genders, immigration pathways, and disabilities. Each participant selected a pseudonym that reflected their identity and enabled confidentiality.

### 2.3. Data Collection

Semi-structured, in-depth interviews were conducted via Zoom. Each interview lasted between 60 and 90 min. Participants from the GTHA were invited to share their migration stories, experiences navigating health and rehabilitation services, sources of community support, and the effects of pandemic restrictions on daily life. Interviewers encouraged reflection, allowing participants to guide the conversation toward issues that were most meaningful to them. All interviews were audio-recorded with participants’ consent and professionally transcribed. Transcripts were returned to participants to allow for review and feedback before coding and analysis.

### 2.4. Data Analysis

Analysis was guided by CDS and CRT, which emphasize examining how power operates in everyday interactions and institutional structures. These frameworks informed how we identified patterns of exclusion and agency within participants’ narratives, interpreting them as products of systemic design rather than personal limitation.

A reflexive thematic analysis was conducted following Braun & Clarke’s six-phase process [29,30]. This approach supported an iterative and interpretive engagement with participants’ narratives. Analysis was guided by the principles of narrative inquiry, which view storytelling as a form of meaning-making within broader social, political, and institutional contexts.

The first three authors began by reading each transcript multiple times to become immersed in the participants’ stories. Early readings focused on understanding each person’s narrative structure and identifying significant events or turning points related to migration, health, and rehabilitation. Initial codes were generated inductively by C.N., K.Y., and K.V., using participants’ own words where possible to stay close to their intended meanings. Codes captured recurring ideas, emotions, and actions related to access, belonging, exclusion, adaptation, and care. NVivo© software, version 14, was used to organize transcripts and codes, which allowed the team to systematically compare patterns across transcripts while maintaining a clear link between coded segments and participants’ original language.

Through iterative team discussions, codes were refined and grouped into interpretive categories that reflected how structural inequities and systems of power shaped everyday experiences of health, rehabilitation, and community life.

Reflexivity was central to the analytic process. The research team discussed their own positionality, interpretive choices, and how their identities, professional backgrounds, and lived experiences informed the analysis. They captured points of tension, insight, and uncertainty, providing a trail of how understanding evolved over time. When differing interpretations emerged, they were explored through collective reflection until a shared meaning was reached.

Several strategies were employed to enhance trustworthiness and transparency. Member checking was incorporated during transcript verification, allowing participants to confirm or clarify how their stories were represented. NVivo provided detailed documentation of analytic decisions, ensuring that themes could be traced back to participants’ words. Peer debriefing within the research team and consultation with the community collaborator further strengthened the credibility and relevance of findings. 

Final themes were organized to highlight participants’ agency, expertise, and creativity alongside their encounters with systemic barriers. The analysis sought to understand how inequities constrained access to health and rehabilitation services and how participants resisted, adapted, and built community-based forms of support. The resulting themes capture both the limitations of formal systems and the relational strategies that sustained participants during the COVID-19 pandemic.

## 3. Results

### 3.1. Participant Overview

Ten participants shared stories that reflected a wide range of migration histories, disability experiences, and connections to health and rehabilitation systems. Participants ranged in age from 20 to 42 years (median = 30) and represented diverse migration pathways from countries in West and Southern Africa, West and Central Asia, Northwestern and Central Europe and the Caribbean. The sample included six women and four men, with eight participants identifying as Black and two as Middle Eastern. Participants had lived in Canada for less than 10 years and were all comfortable communicating in English.

The diversity within the group provided important insight into how systemic inequities are experienced differently depending on immigration status, time in Canada, disability, and access to community supports.

### 3.2. Overview of Findings

Analysis of the ten interviews generated four interconnected themes that describe how Black and racialized immigrants with disabilities navigated health, rehabilitation, and life during the COVID-19 pandemic. The themes reflect both structural barriers and the relational forms of care and resistance that participants built in response: (1) Systems That Made Access Unequal, (2) Feeling Like the “Other of the Other,” (3) Community, Family, and Faith Filling the Gaps Left by Systems, and (4) Disruptions, Adaptations, and Everyday Resistance. Together, these narratives illustrate the social conditions that determined who was seen, supported, or forgotten within health and rehabilitation systems.

#### 3.2.1. Theme 1: Systems That Made Access Unequal

Participants described encountering the same message in different forms: the system was not built for them. Across hospitals, rehabilitation clinics, and community programs, people spoke about invisible rules that quietly decided who could participate and who could not. These rules were embedded in funding policies, program eligibility criteria, and digital or linguistic requirements that assumed everyone had similar resources, supports, and capacities. For many, access depended on factors such as immigration status, language proficiency, or access to technology.

Their stories show how bureaucracy and poor coordination create hidden barriers that are rarely seen as part of healthcare itself. These administrative processes quietly reproduce inequity, even when the services they regulate are described as universal.

The COVID-19 pandemic amplified these inequities. As health and rehabilitation services moved online, participants were expected to navigate digital platforms, complete English-language forms, and understand bureaucratic procedures without assistance. When services resumed, coverage policies and referral systems still favoured those who fit the “ideal” patient profile: permanent residents, technologically literate, and physically independent.

Sara, a 38-year-old woman with a physical disability, immigrated from West Asia five years ago. She relies on an orthotic brace to walk comfortably and had tried several times to have it adjusted through local rehabilitation clinics. During one of those visits, she was told that the service was not covered because of her temporary immigration status.

*“When I went to get my brace adjusted, they told me it wasn’t covered because I was still on a temporary visa. They said, ‘Maybe try later when you become a resident.’ I felt like my pain didn’t count.”*

For Sara, this interaction revealed how coverage policies are tied to immigration status rather than to medical or rehabilitation need. Although her condition required ongoing support, her eligibility for essential equipment depended on bureaucratic categories that placed her care on hold. Her story captures how policies that appear neutral often reinforce inequities for newcomers with disabilities, determining who can access rehabilitation and who is left waiting.

Destiny, a 27-year-old woman with a spinal cord injury from the Caribbean, described how online rehabilitation made her feel excluded. When in-person therapy stopped during the pandemic, her provider suggested continuing sessions virtually.

*“They said I could do my therapy on Zoom, but my Wi-Fi kept cutting out. The exercises weren’t safe for me to do alone, so I just stopped going.”*

For Destiny, what was framed as an innovative solution quickly became another barrier. Her apartment did not have the space or equipment needed for safe movement, and she often worried about injuring herself without supervision. The digital platform further excluded her, showing how virtual rehabilitation can deepen inequities when technology, space, and safety are assumed to be universal.

Molly, a 42-year-old woman from Southern Africa who lives with chronic pain, spoke about how communication shaped her experience in the healthcare system. She described how medical language and professional routines often left her feeling out of place.

*“Sometimes they explain things using medical terms I don’t know. When I ask questions, it feels like I’m wasting their time.”*

Molly said she often prepared questions before appointments, but hesitated to ask them because of how providers responded. She noticed that information was shared as if everyone understood the same words, spoke with the same pace, and had the same expectations of what a “good patient” should do. These interactions left her feeling unsure about when it was appropriate to ask questions and contributed to her decision to remain quiet during appointments. Over time, this shaped how she engaged with health and rehabilitation service providers.

Others described how paperwork and referral systems made it harder to access care. Regina, a 20-year-old woman with a visual impairment who immigrated from West Africa, explained that most health and government forms were not available in accessible formats. She often had to rely on friends to read or complete them, which compromised her privacy and independence. She said she sometimes avoided applying for programs altogether because of the effort involved.

Flash, a 31-year-old man from West Africa, spoke about how referral delays left him without consistent follow-up. He described weeks of waiting for calls that never came and said it often felt as though “something was lost between offices.” Flash’s experience illustrates how poor coordination within referral systems can act as barriers to care, even in the absence of explicit exclusion or denial of services.

Taken together, these stories demonstrate how inequity is built into the everyday operations of health and rehabilitation systems. Access is structured through funding policies, eligibility criteria, communication norms, digital infrastructures, and referral pathways that determine who moves forward and who is left waiting. Participants’ experiences show that barriers are produced through routine administrative practices that continue to reflect colonial histories, racist and ableist assumptions about independence, even when services are described as universal.

#### 3.2.2. Theme 2: Feeling Like the “Other of the Other”

Participants also spoke about what it felt like to never fully belong anywhere. Many described a form of double exclusion where they were both visible and invisible at the same time, seen through stereotypes yet unseen as individuals with knowledge and agency. The phrase “the Other of the Other” captures how they were positioned at the margins of systems that already claimed to be inclusive.

This theme reflects the expectation that individuals conform to dominant cultural and bodily norms to be recognized. Participants’ experiences showed that belonging was conditional, granted only when they could fit into predefined categories of “good immigrant,” “compliant patient,” or “independent disabled person.” When they could not, they were sidelined, dismissed, or made to feel like intruders in spaces that were supposed to include them.

Melanie, a 36-year-old woman from West Africa who uses a wheelchair, described how everyday interactions carried subtle messages about who was seen as competent or intelligent. She said that attitudes often changed the moment people heard her speak.

*“When they hear my accent and see my wheelchair, it’s like double judgment. They talk to me slower, like I don’t understand. Sometimes I just stay quiet.”*

She explained that these moments were rarely overtly hostile, yet they left her feeling invisible and tired of having to prove herself. These biases shaped ordinary conversations, from medical appointments to community settings. Her experience shows how professionalism and politeness can mask deeper hierarchies of credibility, where tone, pacing, and body language quietly communicate who is assumed to belong and who is perceived as less capable.

Tammy, a 36-year-old with a mobility-related disability who immigrated from Central Europe six years ago, described being excluded from a newcomer workshop. The event was held in a community space located on an upper floor with no elevator.

*“I went to a newcomer session, but the space was upstairs with no elevator. I told them, and they said, ‘We didn’t think someone with mobility issues would come.’”*

She recalled feeling embarrassed and angry. What was meant to be a welcoming environment made her feel out of place and invisible. Tammy explained that experiences like this were common, where programs often claimed to be accessible and inclusive but failed to consider who might be left out. Her story reveals how accessibility and inclusion are often imagined through a narrow lens, where newcomers are assumed to be non-disabled, English-speaking, and middle-class. When spaces are designed without accessibility in mind, they send a clear message about who is expected to participate.

Regina described how her credibility was often questioned in medical appointments. She said that even when she introduced herself as the patient, health professionals frequently directed their questions to the person accompanying her.

*“Sometimes they ask my friend questions instead of me, like I’m not there. They don’t see me as the patient. They see my friend as my voice.”*

She explained that the words, tone and body language of health professionals made her feel invisible. She described feeling dismissed and unheard during clinical interactions, particularly when attempting to explain her needs or ask questions.

Some participants also reflected on the emotional impact of this repeated exclusion. Feeling out of place created exhaustion and mistrust that carried into clinical relationships. A few described hesitating to seek healthcare altogether to avoid the frustration and humiliation of being misunderstood or dismissed. This avoidance was an act of self-protection in systems that continually questioned their legitimacy.

These stories reveal that belonging for Black and racialized disabled immigrants was a constant negotiation. Participants were expected to adapt quietly rather than prompting systems to change. In health and rehabilitation systems, inclusion often depends on performance—the ability to appear competent and grateful rather than on the redesign of systems that create exclusion in the first place.

#### 3.2.3. Theme 3: Community, Family, and Faith Filling the Gaps Left by Systems

When formal health and rehabilitation services became difficult to reach, participants turned to the networks they trusted most. Families, neighbours, and faith groups filled the gaps that systems left behind. These connections were foundational, which made daily life possible.

Participants explained that while institutional programs focused on paperwork, eligibility, and protocols, community care focused on relationships and trust. People relied on those who shared their language, faith, and lived experience to get to appointments, interpret medical information, or stay motivated when isolation took hold. In many cases, community became the only reliable infrastructure.

Jack, a 29-year-old man who uses a wheelchair from Southern Africa, described how his church community became the most reliable part of his support network. Transportation was one of his biggest challenges, and he often waited hours for Wheel-Trans rides that never arrived.

*“My church group checked in on me every week. They drove me to appointments when Wheel-Trans didn’t come. Without them, I would have been stuck.”*

He said his church friends never treated their help as charity. It was simply what community did for each other. Their consistency filled the gaps left by unreliable public systems. Jack’s story shows how collective responsibility replaced institutional reliability. While public systems measured access in service counts, his community measured it in relationships and consistency.

Maryam, a 42-year-old woman with an episodic condition, immigrated from Central Asia, spoke about how her neighbour helped her stay active when therapy was cancelled. The two women lived in the same apartment building and often met in the hallway to talk about health and family. When in-person rehabilitation stopped during the pandemic, they decided to keep the exercises going together.

*“My neighbour is a nurse back home. She helped me figure out the exercises from my physiotherapist. It’s like we built our own system.”*

Maryam said these sessions became a source of both physical and emotional support. Her neighbour understood her pain levels and adapted the movements, so they felt manageable. The familiarity of their conversations and shared background made the activities feel safe and even joyful. Their story shows how knowledge and care circulate within communities that health systems often treat as passive. Together, they created a form of rehabilitation that combined clinical understanding with trust, belonging, and connection.

Flash described how WhatsApp groups became a lifeline for both information and connection during the pandemic. When public health updates were unclear and official websites hard to navigate, these community chats became his main source of trusted information:

*“Someone would always post if there was a new clinic or testing site. That’s how I knew where to go. The system didn’t tell us; people told people.”*

He explained that the groups did more than share updates—they created a sense of safety. Members checked in on one another, translated announcements, and shared advice on how to manage appointments and medication. These digital spaces made health information feel human again. Flash’s story shows how communities mobilized technology as a form of collective care and knowledge-making. By turning WhatsApp into an accessible communication tool, participants redefined who counts as an expert and whose knowledge systems matter.

Across interviews, participants emphasized that community support was about survival and dignity. The relationships they built restored a sense of belonging that systems had eroded. In moments of isolation and uncertainty, mutual care became an act of resistance to exclusionary structures.

Participants described this collective care as grounded in reciprocity. Family members who translated forms or helped navigate transportation were not stepping in because systems were generous, but because systems were absent. In these accounts, the labour of driving, explaining, interpreting, and adapting was carried by those already marginalized. Yet these same acts reflected creativity and resistance to being erased, reshaping what health and rehabilitation could look like when it is centred in community rather than confined to institutions.

Together, these stories challenge the idea that equity can be delivered solely through policy reform. They remind us that care is already being practiced every day, in living rooms, places of worship, and group chats. When health and rehabilitation systems fail to meet people where they are, communities build their own infrastructures of access, infrastructures that are relational, culturally grounded, and built on trust.

#### 3.2.4. Theme 4: Disruptions, Adaptations, and Everyday Resistance

The pandemic disrupted nearly every part of participants’ lives, work, community, health and rehabilitation navigation. Services that once provided consistency were suddenly inaccessible or restructured in ways that excluded those who could not meet new expectations. Yet across stories, participants described how they adapted, resisted, and found ways to reimagine health, social and rehabilitation services and daily routines on their own terms. Their actions revealed that resistance can also mean persistence, adaptation, and quiet refusal.

Participants showed that when systems became rigid, they became inventive. Many described repurposing household items for therapy, finding new ways to move safely, or blending healthcare and rehabilitation activities into family routines. These choices reflected both necessity and insight. In the absence of accessible support, people redefined what health and progress looked like for themselves by reshaping it to fit their realities.

Arosa, a 34-year-old woman with a chronic musculoskeletal condition from the Caribbean, spoke about how she adapted her exercises when rehabilitation services were suspended. At first, she found it difficult to stay motivated and missed the sense of structure that therapy provided. Over time, she began inviting her children to join her.

*“I started doing my stretches with my kids, so it felt like play instead of therapy. That helped my mental health, too.”*

She explained that what began as a way to keep up with her program became an important family routine. The movement turned into laughter and connection. Through this small act, she transformed rehabilitation from a medical task into a shared activity that supported her body and her spirit. Arosa’s story shows how care and healing can emerge through relationship, reminding us that rehabilitation is most meaningful when it is part of everyday life rather than separate from it.

Regina refused to accept virtual care as her only option:

*“They said appointments were only virtual, but I kept calling until someone saw me in person. I knew my body better than they did.”*

She said she felt both nervous and determined. Each call was a small act of defiance against a system that prioritized convenience over accessibility. Her persistence challenges the idea that compliance equals good patient behaviour. Regina reasserted her expertise and demanded care that responded to her body rather than to institutional routines. Her story demonstrates resistance and the refusal to disappear from systems that are meant to serve them.

Destiny described learning to let go of guilt for what she could not control:

*“I used to think I was lazy because I couldn’t get everything done. Now I know it’s the system that’s not built for me.”*

Her reflection captures a deeper form of resistance—refusing to internalize blame for structural failure. By naming the system as the problem, Destiny redirects accountability where it belongs.

Across interviews, participants’ actions demonstrated that resistance is an active, ongoing critique of systems that expect people to adjust to inaccessibility. These everyday acts of resistance, making care collective, insisting on being heard, and rejecting guilt, reveal how people generate their own frameworks for health and dignity within exclusionary structures.

Participants’ stories also highlight the limits of institutional definitions of health and rehabilitation. Progress was often measured by independence or compliance, yet participants’ experiences showed that health also depends on community, creativity, and control over one’s time and body. Their adaptations were examples of redesign—creating accessible ways of living that systems had failed to provide.

Together, these stories point to a critical insight: people are redesigning health and rehabilitation services around interdependence, relational accountability, and lived expertise. In doing so, they expose how systems could change to make structures more responsive.

### 3.3. Summary

Across these stories, participants revealed that what limited them most were the systems that determined who could access care, who was believed, and who was forgotten. Each theme exposed how those systems operate—through policies that tie coverage to immigration status, through assumptions about language and technology, through service models that privilege independence, and through professional norms that overlook relational and cultural forms of care.

Participants’ experiences showed that exclusion was a product of design. Health and rehabilitation programs often relied on standardized procedures that assumed English fluency, stable housing, permanent residency, and non-disabled independence. When those assumptions failed, people were left to bridge the gaps themselves. They did this by mobilizing their own networks, being creative, and resisting the status quo.

Participants’ actions and insights demonstrated another way of understanding care: one rooted in connection and reciprocity. Their stories remind us that health is not produced solely in clinics or policies but in the everyday acts of people who sustain one another despite institutional neglect.

Taken together, these findings point to a clear message. People are already practicing the kind of health and rehabilitation care that institutions aspire to, care that is inclusive, responsive, and grounded in community. Their knowledge and resistance challenge systems to catch up. True equity will not come from asking individuals to adapt to inaccessible structures, but from transforming those structures to reflect the realities, strengths, and wisdom of the people they serve.

## 4. Discussion

### 4.1. Health and Rehabilitation as Systems of Inequity

The findings from this study reveal that inequities within health and rehabilitation are not merely legacies of the pandemic—they are inherent features of how these systems continue to operate today. Participants’ stories reveal how the very structures intended to provide care continue to reproduce exclusion through policies, procedures, technologies, and attitudes that assume a narrow definition of who belongs. These systems remain organized around ideals of independence, efficiency, and neutrality that fail to reflect the realities of disabled people’s lives.

In today’s health and rehabilitation systems, access is still treated as a personal responsibility rather than a collective right. Policies that tie coverage to immigration status, eligibility criteria that privilege full-time employment, and digital platforms that presume reliable internet all reflect assumptions about stability, language, and ability. Similar dynamics are described in global disability research where digitalization reinforced existing inequalities [6,31]. These assumptions mirror what continues to dominate professional practice: the expectation that patients will self-manage and communicate in standardized ways. Participants’ accounts from Sara’s denied orthotic adjustment to Destiny’s unsafe virtual therapy expose how these assumptions translate into material barriers that shape who can benefit from rehabilitation and who cannot. While tele-rehabilitation and electronic systems have been praised for expanding access, they also reinforce ableist and racialized norms when they are designed without attention to cultural and linguistic diversity. Participants’ experiences with online health and rehabilitation services show how “innovation” can deepen exclusion when it assumes everyone has access to the same tools, space, and safety.

At the same time, the professional culture of health and rehabilitation continues to reward independence and individual progress as markers of success. These measures, which remain central to clinical practice and outcome reporting, obscure how health is shaped by social relationships, housing, immigration status, and community connection. Participants like Regina and Melanie described being positioned as less credible and competent because their ways of communicating did not match these clinical expectations. Their stories reveal how contemporary health and rehabilitation systems still reflect a biomedical model that privileges the voices of professionals over those of patients, and standardization over flexibility.

Participants’ accounts also highlight how communication within health and rehabilitation settings functions as a site of power rather than a neutral exchange. Clinical interactions are shaped by professional norms that privilege particular ways of speaking, processing information, and demonstrating competence, often aligned with whiteness, normative standards of ability, and fluency in dominant languages [15,16,19]. When information is delivered at a fixed pace and through standardized language, responsibility for understanding is implicitly shifted onto patients rather than shared by systems. This dynamic has been identified in critical disability and race scholarship as a form of ableism and linguistic bias that undermines participation and credibility within healthcare encounters [15,19]. Molly’s experience reflects how these norms shape access to information and how individuals engage with care, including decisions to remain silent or disengage when communication feels inaccessible or dismissive.

Participants’ reflections also highlight a growing tension in health and rehabilitation today—between professional ideals of individualized recovery and the realities of collective survival. When participants-built networks of care, adapted exercises with family, or relied on faith-based support, they demonstrated models of health that already embody people-centred practice. These examples challenge the notion that inclusion requires more efficiency or innovation. Instead, they show that inclusion begins with relationships, trust, and accountability, values that continue to be undervalued in current system reform efforts.

In interpreting these findings, it becomes clear that health and rehabilitation systems remain at a crossroads. On one hand, policy conversations about equity, digital transformation, and accessibility are more visible than ever. On the other hand, participants’ experiences show how these reforms risk reproducing the same exclusions if they fail to address how race, disability, and immigration status shape access and belonging. The stories from this study remind us that transforming health and rehabilitation is about redesigning the conditions under which access becomes meaningful, where care reflects the lives and communities it is meant to serve.

### 4.2. Community, Resistance, and Redefining Care

Participants’ stories revealed that when formal health and rehabilitation systems became difficult to reach, people built their own. Family members, neighbours, faith communities, and informal networks became the true infrastructure of care. Similar community-driven responses have been documented across contexts [4,6,7,22]. These forms of support were essential. They filled the spaces left by systems designed around efficiency and standardization rather than relationship and belonging.

In today’s health and rehabilitation systems, care is often organized through metrics, performance targets, and time-limited appointments. These approaches rarely reflect the ongoing, relational, and collective ways that participants sustained health. Jack’s church community, which provided transportation, Maryam’s neighbour, who helped her complete physiotherapy exercises, and Flash’s WhatsApp network, which shared information about clinics, all demonstrate how health and rehabilitation are already practiced beyond institutions. These actions show how community connection functions as a form of expertise, one that recognizes that health is lived collectively, not individually.

Such community-based strategies represent forms of resistance to systems that continue to privilege individualism and clinical authority. Participants refused to wait for inclusion; they created it. This is particularly relevant in contemporary rehabilitation, where digital transformation, staffing shortages, and funding cuts have intensified the pressure on individuals to self-manage. Yet the stories in this study remind us that people have always managed collectively. In doing so, they expose the limits of current models that measure success by independence and compliance rather than connection and care.

This redefinition of care resonates with broader discussions in public health and rehabilitation about the need for community-driven and culturally grounded models of practice [32,33]. Participants’ networks embodied these models long before they were named in policy. They demonstrated how trust, reciprocity, and local knowledge can bridge gaps left by formal systems. These findings suggest that people-centred care is already happening—just not always in the places institutions are looking.

Participants’ actions also complicate the language of “resilience,” which is often used in health research to celebrate endurance under inequitable conditions. What they demonstrated was resistance, the creative and collective work of redefining what counts as rehabilitation and who holds expertise. When Arosa integrated her therapy into family routines, or Regina refused to accept virtual appointments as her only option, they were not simply adapting; they were challenging the underlying assumptions of the system. Their strategies point toward a version of rehabilitation that values flexibility, interdependence, and shared problem-solving qualities that remain underdeveloped in health system reform.

Drawing on intersectional scholarship, Sami Schalk [34] emphasizes how interlocking systems of racism, ableism, and sexism shape lived experience beyond singular identity claims. This approach directs attention to power relations that structure whose bodies, needs, and knowledge are rendered visible or marginalized. Bell’s critique of white disability studies [35] further demonstrates how analyses that detach disability from race and ethnicity reproduce partial understandings, limiting how actions are interpreted and whose experiences are taken seriously within social and healthcare systems.

These examples have immediate relevance for health and rehabilitation practice today. They show that people already build care systems that are culturally familiar, flexible, and community-led. The challenge for health and rehabilitation professionals is not to “teach” these models but to learn from them. Incorporating community networks, faith leaders, and peer support into system design could strengthen trust and accessibility while addressing gaps in continuity of care. Doing so would align with ongoing efforts to build people-centred and community-driven systems that move towards genuine co-design.

In this way, participants’ everyday acts of care and refusal become blueprints for the kind of health and rehabilitation systems that are needed today, ones that honour lived expertise, share responsibility, and recognize that equity is built through relationships, not efficiency.

### 4.3. Toward People-Centred Systems

The stories in this study reveal what people-centred systems already look like in practice. Participants described forms of care grounded in trust and shared responsibility. Their experiences suggest that when health and rehabilitation are shaped around people’s lives rather than institutional routines, access and belonging become possible.

Current reforms in Canada emphasize digital care, interprofessional collaboration, and system efficiency. These are valuable directions, but participants’ narratives remind us that people-centeredness depends on something less technical and more relational. National public-health scholarship reinforces this need for relational accountability. *Measuring What Counts* offers practical “equity prompts” for pandemic preparedness, demonstrating how governance, data collection, and workforce planning can embed justice and inclusion as performance measures [14]. Integrating such frameworks would help operationalize the people-centred approaches participants described—approaches that grow from being seen, listened to, and understood within one’s cultural, linguistic, and community context. Sara’s and Destiny’s stories illustrate what happens when those relationships are missing: policies and technologies that appear neutral can exclude people whose lives do not fit the system’s template.

Health and rehabilitation today still define success through administrative outcomes such as service volumes or wait-time reduction. Participants’ accounts point to different measures, trust, communication, and continuity that more accurately reflect what matters to people navigating exclusion. Evaluating systems through these relational indicators would provide a clearer picture of equity than numerical targets ever could.

Building people-centred systems also means recognizing that communities already carry much of the health work that institutions claim to deliver. Families organize transportation, translate information, and provide motivation when formal services end. Faith leaders and neighbours act as connectors between professional and everyday knowledge. Supporting these networks through sustained funding, training, and policy alignment would make health and rehabilitation services more responsive and sustainable.

Partnerships with community organizations must begin early, before programs are designed or policies written. Participants’ networks demonstrate that collaboration works best when decision-making power and accountability are shared. When lived experience informs planning, systems become more adaptive to cultural differences and changing social conditions.

People-centred systems also require a re-examination of professional authority. Expertise in health and rehabilitation does not reside solely in credentials; it also lives in communities that have learned to navigate barriers creatively. Recognizing community knowledge as equal to professional knowledge can reshape how programs are delivered and evaluated. For rehabilitation practitioners, this means approaching each interaction as a partnership and understanding adaptation as shared work rather than patient compliance.

The participants in this study showed that equity begins with relationships of respect and accountability. Their experiences align with a growing movement in health care that treats inclusion as an everyday practice—built through listening, reciprocity, and humility. People-centred systems emerge when institutions are willing to learn from the people they serve and allow those relationships to guide policy, training, and care delivery.

## 5. Conclusions

This study contributes a detailed account of how Black and racialized immigrants with disabilities experienced health and rehabilitation systems during the COVID-19 pandemic. The stories shared by participants bring forward experiences that remain largely invisible in Canadian health research. By centring lived experience, the study offers a view of health and rehabilitation as social systems that shape everyday life rather than as neutral sites of service delivery.

### Limitations and Moving Forward

There are limits to what this study can claim. All participants lived in an urban region and were comfortable communicating in English, which means that the perspectives of non-English-speaking immigrants or those in rural or northern communities are missing. The online format made participation possible during periods of physical distancing but may have excluded those with limited digital access. As with most qualitative work, the aim was not to generalize but to offer insight into how systems operate and how people experience and challenge them.

Future research could extend this work in several directions. Studies in other provinces and in rural or northern regions could explore how geography and resource availability shape access to health and rehabilitation services. Research that includes non-English-speaking immigrants would deepen understanding of linguistic barriers. Longitudinal studies could also follow participants over time to see how relationships with health and rehabilitation systems evolve as immigration status and digital infrastructure change. Given that many participants identified as Black, future research should explicitly examine anti-Black racism as a distinct structuring force within health and rehabilitation systems. While this study addressed intersecting forms of racism, ableism, and immigration status, a focused anti-Black racism analysis could more precisely explore how Blackness shapes credibility, clinical encounters, and access to healthcare and rehabilitation services. Attending to anti-Black racism as its own analytic frame is essential for advancing equitable health and rehabilitation research.

Moving forward, the insights from this study can guide both practice and policy. For practitioners, the findings highlight the importance of listening deeply, building relationships, and seeing patients as partners in knowledge. For policymakers, they emphasize the need to design systems that value lived expertise and that fund the community networks already doing much of the health and rehabilitation work on the ground. Building equitable systems begins with accountability—acknowledging how current structures exclude and working with the people most affected to redesign them. 

The participants in this study have already begun that work. Through their stories, they offer a vision of health and rehabilitation that is people-centred, collaborative, and grounded in care that extends beyond the healthcare provider’s clinic. Their experiences remind us that equity is a practice sustained through connection and that change becomes possible when institutions are willing to learn from those who have always found ways to make systems work for each other.

## Data Availability

The datasets presented in this article are not readily available due to ethical restrictions and participant confidentiality. Requests to access the datasets should be directed to correspondence author.

## References

[1] Underwood K., van Rhijn T., Balter A.-S., Feltham L., Douglas P., Parekh G., Lawrence B. (2021). Pandemic Effects: Ableism, Exclusion, and Procedural Bias. J. Child. Stud..

[2] Caleb A.M., Gallin S. (2021). Policies of Exclusion: The Impact of COVID-19 on People with Disabilities. St. Louis Univ. J. Health Law Policy.

[3] Yoshida K.K., Schormans A.F., Niles C., Mahipaul S. (2021). COVID-19 Amplifies the Complexity of Disability and Race. https://medicalxpress.com/news/2021-04-covid-amplifies-complexity-disability.html.

[4] Juvva S., Nayar M., Sinha R. (2022). Living in the Margins: Disability and the Pandemic. Habitus J. Sociol..

[5] Shakespeare T., Ndagire F., Seketi Q.E. (2021). Triple Jeopardy: Disabled People and the COVID-19 Pandemic. Lancet.

[6] van Holstein E., Wiesel I., Bigby C., Gleeson B. (2022). More-than-Care: People with Intellectual Disability and Emerging Vulnerability during Pandemic Lockdown. Trans. Inst. Br. Geogr..

[7] Niles C.A., Hande M.J., Fudge Schormans A., Porch W., Mahipaul S., Anderson J., Yoshida K. (2025). What and Who Are “Essential”? A Disability Justice Perspective on COVID-19 Measures and the Diverse Disability Communities in Ontario. Stud. Soc. Justice.

[8] Oguamanam C. (2020). Legal Redress for Persons with Disabilities in Pandemic Situation. https://ssrn.com/abstract=3995651.

[9] Gill M. (2020). A Disability-Inclusive Response to COVID-19. https://unsdg.un.org/sites/default/files/2020-05/Policy-Brief-A-Disability-Inclusive-Response-to-COVID-19.pdf.

[10] Statistics Canada (2018). Canadian Survey on Disability, 2017: A Demographic, Employment and Income Profile of Canadians with Disabilities Aged 15 Years and Over (Catalogue No. 89-654-X2018002). https://www150.statcan.gc.ca/n1/en/daily-quotidien/181128/dq181128a-eng.pdf.

[11] Employment and Social Development Canada (2021). Disability Inclusion and Accessibility. https://www.canada.ca/content/dam/esdc-edsc/documents/corporate/reports/esdc-transition-binders/2021/08-disability-and-inclusion-and-accessibility-1025-en-pr.pdf.

[12] Komeiha M., Artyukh I., Ogundele O.J., Zhao Q.J., Massaquoi N., Straus S., Razak F., Hosseini B., Persaud N., Mishra S. (2025). Unveiling the impact: A scoping review of the COVID-19 pandemic’s effects on racialized populations in Canada. Int. J. Environ. Res. Public Health.

[13] Obress L., Berke O., Fisman D.N., Tuite A.R., Greer A.L. (2022). Sporadic SARS-CoV-2 cases at the neighbourhood level in Toronto, Ontario, 2020: A spatial analysis of the early pandemic period. CMAJ Open.

[14] Haworth-Brockman M., Betker C., Webb S. (2025). Measuring What Counts: Equity Prompts for Public Health Preparedness and Resilience.

[15] Erevelles N. (2011). Disability and Difference in Global Contexts: Enabling a Transformative Body Politic.

[16] Dossa P. (2009). Racialized Bodies, Disabling Worlds: Storied Lives of Immigrant Women.

[17] Crenshaw K. (1989). Demarginalizing the intersection of race and sex: A Black feminist critique of antidiscrimination doctrine, feminist theory and antiracist politics. Univ. Chic. Leg. Forum.

[18] Frank K. (2022). Difficulties accessing health care in Canada during the COVID-19 pandemic: Comparing individuals with and without chronic conditions. Health Rep..

[19] Stienstra D. (2018). Canadian disability policies in a world of inequalities. Societies.

[20] Barden O., Bê A., Pritchard E., Waite L. (2023). Disability and social inclusion—Lessons from the pandemic. Soc. Incl..

[21] Kett M., Cole E., Twigg J. (2018). Disability and Climate Resilience: A Literature Review.

[22] Ned L., McKinney E.L., McKinney V., Swartz L. (2022). Experiences of vulnerability of people with disabilities during COVID-19 in South Africa. S. Afr. Health Rev..

[23] Connon I.L.C., Crampton A., Dyer C., Hu R.X. (2024). Social disability as disaster: Case studies of the COVID-19 pandemic on people living with disabilities. Soc. Sci..

[24] Oliver M. (1990). The Politics of Disablement: A Sociological Approach.

[25] Slater J. (2013). Constructions, Perceptions and Expectations of Being Disabled and Young: A Critical Disability Perspective. Ph.D. Thesis.

[26] Riessman C.K., Huberman A.M., Miles M.B. (2002). Narrative analysis. The Qualitative Researcher’s Companion.

[27] Riessman C.K. (2005). Narrative analysis. Narrative, Memory & Everyday Life.

[28] Riessman C.K. (2008). Narrative Methods for the Human Sciences.

[29] Braun V., Clarke V. (2006). Using thematic analysis in psychology. Qual. Res. Psychol..

[30] Braun V., Clarke V. (2021). Thematic Analysis: A Practical Guide.

[31] Eriksson M., Nilsson E.M., Lundälv J. (2023). A scoping review of research exploring working-life practices of people with disabilities during the COVID-19 pandemic. Scand. J. Disabil. Res..

[32] Minkler M., Wallerstein N. (2011). Community-Based Participatory Research for Health: From Process to Outcomes.

[33] Israel B.A., Eng E., Schulz A.J., Parker E.A. (2013). Methods for Community-Based Participatory Research for Health.

[34] Schalk S. (2018). Bodyminds Reimagined: (Dis)Ability, Race, and Gender in Black Women’s Speculative Fiction.

[35] Bell C.M., Davis L.J. (2006). Introducing White Disability Studies: A Modest Proposal. The Disability Studies Reader.

